# Work‐related injuries and illnesses and their association with hour of work: Analysis of the Oregon construction industry in the US using workers’ compensation accepted disabling claims, 2007‐2013

**DOI:** 10.1002/1348-9585.12118

**Published:** 2020-03-20

**Authors:** Liu Yang, Adam Branscum, Ellen Smit, David Dreher, Karen Howard, Laurel Kincl

**Affiliations:** ^1^ College of Public Health and Human Sciences Oregon State University Corvallis OR USA; ^2^ Public Health Division Oregon Health Authority Portland OR USA; ^3^ Central Services Division Oregon Department of Consumer and Business Services Salem OR USA

**Keywords:** accepted disabling claim, construction industry, hour of work, workers’ compensation, work‐related injury and illness

## Abstract

**Objectives:**

This study aimed to characterize injuries and illnesses among construction workers in the State of Oregon in the US and examine the association between injury frequency and severity with hour of work by using Workers’ Compensation (WC) accepted disabling claims data in the construction industry from 2007 to 2013.

**Methods:**

Injury frequency, rate, medical cost, and lost work days were analyzed by year, demographics, employment, injury nature, and temporal factors including hour of work. Multiple linear regression models were used to quantify adjusted associations between hour of work and medical cost and lost work days (indicating injury severity).

**Results:**

There were a total of 12 222 disabling claims in the Oregon construction industry. The average annual injury rate was 2.21 per 100 workers. Both the count and rate of disabling claims decreased during the study period. Male workers and young workers had higher injury rates, while medical cost and lost work days increased for older workers. Injuries occurring at night were more severe. The distribution of claims frequency by hour of work was bimodal, with peaks in the 4th and 8th hour. Compared with the first hour of work, the 5th and 13th hours corresponded to significantly more severe injuries and illnesses.

**Conclusions:**

This study identified the burden and distribution of work‐related injuries and illnesses in the Oregon construction industry. Continued intervention efforts should target certain subpopulations (eg, young workers) and certain working time periods (eg, mid‐ and end‐shift) to protect construction workers’ safety and health.

## 1 INTRODUCTION

The construction industry has consistently been a high‐risk industry worldwide. Compared with other industrial countries, the United States (US) had fairly high fatal and non‐fatal injury rates.^1^ Nationally, there were approximately 1000 work‐related fatalities annually, corresponding to an occupational fatality rate of 9.5 per 100 000 full‐time equivalent workers (FTEs). This is almost three times higher than the fatality rate for all private industries combined in the US The national rate of non‐fatal occupational injuries and illnesses for construction workers was 3.0 per 100 FTEs in 2017. In the State of Oregon, the non‐fatal work‐related injury and illness rate in the construction industry was 1.6 times higher than the national rate in 2017.^2^

Work‐related injuries and illnesses are preventable. Understanding the burden of workplace injuries and illnesses and determining contributing factors are critical in guiding the development of prevention strategies. Workers’ Compensation (WC) claims data have been used to quantify work‐related injuries and illnesses and to identify risk factors in different countries, including the US.^3^, ^4^, ^5^ There has been no analysis of recent Oregon WC claims data specifically for the construction industry to our knowledge.

Temporal exposures at work have been shown to impact injury risk. A few existing studies indicate that the distributions of injuries by time into the work shift (ie, hour of work) follow certain patterns. For example, studies on manufacturing and construction industries suggest that more injuries occurred in the first half of an 8‐hour work shift, especially within the first 3 hours of work.^6^, ^7^, ^8^ We further hypothesize that injury severity is also related to hour of work. However, no existing study has reported on this association to the best of our knowledge. The Oregon WC claims data are particularly well positioned for this analysis because it includes variables on injury characteristics and associated costs, claimants’ demographics and employment status, as well as temporal factors such as the time of injury and the start time of the worker's shift before the injury.

This study aimed to analyze work‐related injury and illness in the Oregon construction industry. Injury frequency and rate by demographics, employment, and temporal factors were determined. Injury severity, as indicated by compensated medical cost and lost work days, was examined in relation to hour of work.

## 2 METHODS

### 2.1 Data source

Oregon employers are required by state law to provide workers’ compensation (WC) coverage to their employees, including hourly and part‐time employees.^9^ A WC claim is considered as “accepted disabling” if it was accepted by insurers and involved missing 3 or more days of regularly scheduled work, overnight hospitalization, likely permanent disability, or death. The insurer is required to report accepted disabling claims to the Workers’ Compensation Division of the Oregon Department of Consumer and Business Services (DCBS), Oregon's largest regulatory and consumer protection agency. The Workers’ Compensation Division reviews claims and manually codes them using the following standard classification systems: employer's industry by the North American Industry Classification System (NAICS), worker's occupation by the Standard Occupational Classification System (SOC), and incident characteristics and circumstances by the Occupational Injury and Illness Classification System (OIICS). Detailed information on Oregon WC claims and DCBS’s data processing procedure is described elsewhere.^10^

De‐identified WC accepted disabling claims for construction workers (NAICS code: 23*) in the state of Oregon from 2007 to 2013 were obtained from the DCBS through a research collaboration in January 2015. The dataset reflects only disabling claims determined as accepted by 2013. Claims can take years to be fully accepted. We found that the number of claims used in this study was approximately 21% underestimated for 2013 as opposed to an average of 0.6% for the previous years (2007‐2012) when compared with DCBS’s more recent data. We retained the 2013 data in this study despite the limitation, considering that it could provide useful information on work‐related injuries and illnesses in 2013, which was otherwise unavailable for this study.

The dataset contains the following variables that were used in this study: injury date and time, time the claimant started the work shift in which the injury occurred, worker's demographics and employment information, injury nature, compensated medical cost, and temporary disability days (ie, lost work days during medical treatment) due to the injury.

To calculate rates, employment information (denominators) was obtained from the Oregon Current Employment Estimates (CES), publicly available via the State of Oregon Employment Department (OED).^11^ The OED publishes total non‐farm employment, including part‐time and hourly workers by year and industry. The CES data do not include employment breakdowns by demographics. We used the US Census Bureau Quarterly Workforce Indicators (QWI) data to calculate the proportions of employment by gender and by age group. The proportions were applied to the CES data to obtain employment estimates by gender and age. We did not use the QWI data directly because the absolute employment numbers are distorted using a system of multiplicative noise infusion for the sake of confidentiality protection.^12^ This method has been used in a previous study.^10^

The Oregon State University Institutional Review Board ethics review of the study was determined to be exempt under the regulation set forth by the US Department of Health and Human Services 45 CFR 46 (Review#: 7807).

### 2.2 Measurements

To estimate the hours into the work shift when an injury occurred, the variable “hour of work” was created by calculating the difference $T_{I}-T_{S}$ between the time of injury ($T_{I}$) and the time the claimant started the work shift when the injury occurred ($T_{S}$), with an adjustment by adding 24 to the difference when $T_{I}<T_{S}$. Both $T_{I}$ and $T_{S}$ were coded from 0 to 23 hours. For this study, the hour of work ranged mostly from 1 to 13 hours (97% of all cases). The first hour corresponds to an injury occurring during the first hour of the work shift, and, in general, the “*k*th hour” corresponds to an injury occurring between hours *k‐1* and *k* of the work shift. Claims with hour of work >13 were aggregated into one category due to the small counts in this range.

Information on claimant's occupation, industry sector, and injury nature was originally coded with six‐digit standard codes (SOC, NAICS, or OIICS (v1.01)). To reduce the number of categories, fewer coding digits were used and categories with less than 2% of claims were combined into one category.

More than 97% of all accepted disabling claims were closed by insurers and thus included complete information on compensated medical cost and lost work days. Medical costs and weekly wages were standardized to the 2013 US dollar based on inflation information from the Bureau of Labor Statistics (BLS) website.^13^

### 2.3 Analysis

The number of claims was counted by year, demographics (age group and gender), employment (construction sector and occupation), injury nature, and temporal factors (work shift start time, assigned shift length, injury time, and hour of work). The injury rate was calculated by year, age group, gender, and construction sector.

Claims were enumerated for each hour of work. The relative mean number of claims by each hour of work was compared using Poisson models. Claims data were also cross‐tabulated by hour of work and injury nature, age group, and occupation and construction sector. Chi‐squared tests examined whether these factors were associated with hour of work.

Average compensated medical cost and average days of lost work were calculated by year, demographics, employment, injury nature, and temporal factors. Linear regression models were used to relate medical cost and lost work days to hour of work. Multiple regression models were adjusted for age, gender, construction sector, occupation, injury nature, weekly wage, and injury time, which were selected based on previous literature and/or to include potentially confounding variables. The response variables (medical cost and lost work days) were log transformed and statistical inference focused on median costs, median number of lost work days, and estimating relative medians.

All estimates were calculated for fatal and non‐fatal injuries combined. No separate analysis of fatal injuries was presented in this study due to the small number of cases. Fatal injuries were excluded from analyses of compensated medical cost and lost work days as no such information exists. Sensitivity analyses were conducted by analyzing the data with and without the 2013 cases to investigate possible impacts due to underestimation in the 2013 data.

All statistical analyses were performed using R version 3.5.1. A two‐sided 95% confidence level was applied.

## 3 RESULTS

### 3.1 Demographics and claims characteristics

There were 12 222 accepted disabling WC claims in the Oregon construction industry from 2007 to 2013. Thirty‐six claims were fatal injuries, accounting for 0.3% of the claims. The majority of the claims involved traumatic injuries (91.7%, excluding 12.5% claims with missing information on injury nature).

There was a 56% decrease in the number of claims from 2007 to 2013 (Table 1). The vast majority of claims were for male workers. The claimants were 16‐79 years of age at the time of injury, with a mean of 39.4 (SD: 11.6) years. Workers aged 25‐54 had the most injuries (78%). The claimants earned an average weekly wage of $815.60 (ranging from $1.1 to $7091, in 2013 US dollars). Most of the claimants were construction trades workers (66.0%) and worked in the “specialty trade contractors” sector (62.6%).

**Table 1:** Workers’ compensation accepted disabling claims frequency, rate, medical cost, and lost work days by selected factors; Oregon construction industry, 2007‐2013

	Number of claims (%, excluding missing data)	Rate per 100 workers (95% CI)	Rate ratio (RR) (95% CI)	Average medical cost (2013 US $)	Average lost work days
Total	12 222 (100.0)	2.21 (2.17, 2.25)	/	11 975	80.5
Year					
2007	2840 (23.2)	2.73 (2.63, 2.83)	1	11 473	81.2
2008	2184 (17.9)	2.32 (2.22, 2.42)	0.85 (0.81,0.90)	12 587	85.5
2009	1556 (12.7）	2.10 (2.00, 2.21)	0.77 (0.73, 0.82)	13 776	94.7
2010	1413 (11.6)	2.09 (1.98, 2.20)	0.77 (0.72, 0.82)	13 337	86.1
2011	1446 (11.8)	2.11 (2.00, 2.22)	0.77 (0.73, 0.83)	12 917	83.9
2012	1523 (12.5)	2.18 (2.07, 2.29)	0.80 (0.75, 0.85)	11 481	72.5
2013	1260 (10.3)	1.70 (1.61, 1.79)	0.62 (0.58, 0.67)	7510	50.2
Gender					
Female	508 (4.2)	0.51 (0.47, 0.55)	1	12 259	90.6
Male	11 714 (95.8)	2.59 (2.54, 2.64)	5.10 (4.67, 5.58)	11 962	80.1
Age group					
16‐24	1301 (10.7)	2.84 (2.68, 2.99)	1.12 (1.05, 1.19)	7329	48.0
25‐34	3293 (27.1)	2.54 (2.45, 2.63)	1	10 803	72.0
35‐44	3324 (27.3)	2.35 (2.27, 2.43)	0.93 (0.88, 0.97)	12 298	84.1
45‐54	2869 (23.6)	2.12 (2.05, 2.20)	0.84 (0.80, 0.88)	13 690	96.4
55‐64	1278 (10.5)	1.60 (1.52, 1.69)	0.63 (0.59, 0.67)	14 341	87.1
>65	108 (0.9)	0.52 (0.43, 0.63)	0.21 (0.17, 0.25)	15 319	96.5
Construction sector					
Residential building construction	1666 (13.6)	2.15 (2.05, 2.25)	1.35 (1.27, 1.44)	11 724	81.2
Non‐residential building construction	995 (8.1)	1.73 (1.63, 1.84)	1.09 (1.01, 1.17)	12 192	81.2
Heavy and civil engineering construction	1908 (15.6)	2.78 (2.65, 2.90)	1.74 (1.64, 1.85)	13 415	77.6
Building foundation and exterior contractors	2342 (19.2)	3.46 (3.32, 3.60)	2.17 (2.05, 2.30)	12 203	83.0
Building equipment contractors	2435 (19.9)	1.59 (1.53, 1.66)	1	11 094	75.1
Building finishing contractors	1852 (15.2)	2.29 (2.19, 2.40)	1.44 (1.35, 1.53)	11 722	85.1
Other specialty trade contractors	1024 (8.4)	2.14 (2.01, 2.27)	1.34 (1.25, 1.44)	11 578	82.3
Shift start time					
06:00‐17:00	8159 (95.2)	/	/	11 857	77.7
18:00‐05:00	411 (4.8)	/	/	13 353	89.7
Assigned shift length					
8 h	2540 (88.6)	/	/	11 544	78.9
10 h	225 (7.8)	/	/	10 874	66.5
Other	103 (3.6)	/	/	10 493	71.1
Injury time					
06:00‐17:00	8428 (93.7)	/	/	11 950	77.3
18:00‐05:00	570 (6.3)	/	/	13 580	83.0

Abbreviation: CI, confidence interval.

The vast majority started work in the morning, with 90% of claimants starting their shifts between 6:00 am and 8:00 am. About 24% of the claims had information on the scheduled hours for the work shift, of which approximately 90% worked an 8‐hour shift. More than 80% of injuries occurred between 8:00 and 14:00 (excluding 26.3% of claims with missing injury time information).

### 3.2 Injury rate

Oregon construction workers had an average annual accepted disabling claims rate of 2.21 per 100 workers (95% confidence interval (CI) 2.17‐2.25) from 2007 to 2013 (Table 1). There was a clear declining trend of injury rate during the study period, from 2.73 per 100 workers in 2007 to 1.70 per 100 workers in 2013. Building equipment contractors had the lowest annual injury rate of 1.59 per 100 workers, whereas building foundation and exterior contractors had the highest rate of 3.46 per 100 workers.

Male workers had an injury rate of 2.59 per 100 workers (95% CI: 2.54‐2.64), which is more than five times the rate in female workers (95% CI: 4.67‐5.58). Considering the different age groups, young workers experienced the highest injury rate (2.84 per 100 workers) and the older age groups had lower injury rates. The oldest age group (aged >65) had an injury rate that was only 21% of the rate for the age group from 25 to 34.

### 3.3 Injury distribution by hour of work

A total of 7382 WC claims (60.4%) contained complete data on hour of work. There were no important differences between claims with and without complete data in terms of claimants’ demographics, employment status, and temporal factors (data not shown).

**Figure 1:**
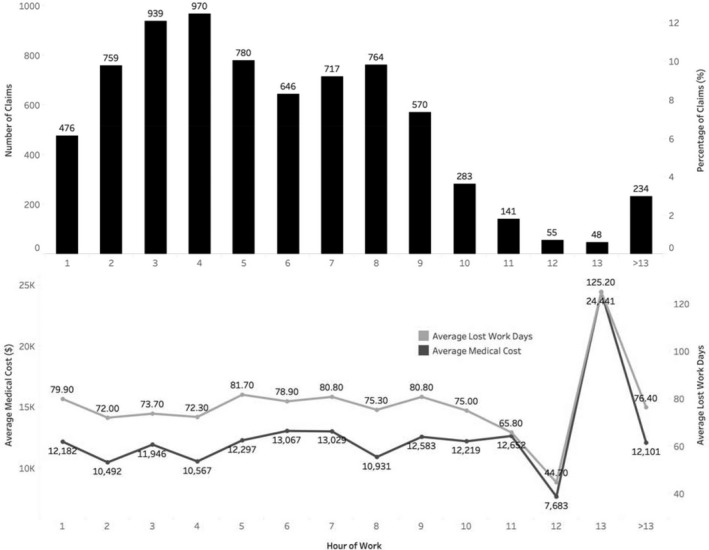
Workers’ compensation accepted disabling claims frequency and percentage, average compensated medical cost, and average lost work days by hour of work, Oregon construction industry, 2007‐2013

Claims were enumerated for each hour of work (Figure 1). There were 6051 claims (82.0%) that occurred within the first 8 hours of work. The number of claims increased from the shift start time to the 4th hour of work, when the frequency was the highest (970 cases, 13.1%). Compared to the first hour of work, the number of injuries and illnesses reported in the 4th hour doubled (relative mean 2.04; 95% CI: 1.83‐2.28). The number of claims dropped in the 5th and 6th hour of work, and then increased to a smaller peak in the 8th hour. Compared with the 6th hour of work, the average numbers of injuries and illnesses in the 7th and 8th hour were statistically higher (relative mean: 1.11 and 1.18; 95% CI: 1.00‐1.31 and 1.07‐1.31, respectively). The numbers of injuries and illnesses after the 8th hour of work dropped substantially.

Stratifying claims by injury nature, similar injury distribution patterns were observed for most injury nature categories (Table 2). In general, there was an increasing trend in claims frequency in the first 4 hours of work, followed by a substantial decrease usually in the middle of a regular 8‐hour shift, and then an increase to another peak toward the end of the work shift. Traumatic injuries tended to occur more often in the first 4 hours. Unlike other injury types, a higher portion of digestive diseases and disorders occurred in the 5th to 6th hour of work, although the total incidence of digestive diseases and disorders was relatively small. A chi‐squared test indicated that injury nature was significantly associated with the hour of work when an injury occurred (*P*‐value: .003).

**Table 2:** Injury nature by hour of work for workers’ compensation accepted disabling claims in the Oregon construction industry, 2007‐2013

Hour of work	Injury Nature (column, ie, injuries per cent within each injury nature category)								
	Traumatic injuries to bones, nerves, spinal cord	Traumatic injuries to muscles, tendons, ligaments, joints, etc	Open wounds	Surface wounds and bruises	Multiple traumatic injuries and disorders	Other traumatic injuries and disorders	Digestive system diseases and disorders	Musculoskeletal system and connective tissue diseases and disorders	Other injuries/disorders/diseases/conditions
1	44 (5.6)	156 (7.0)	35 (5.6)	16 (4.6)	115 (7.7)	23 (4.4)	3 (2.0)	11 (8.7)	13 (8.4)
2	84 (10.6)	246 (11.1)	65 (10.4)	29 (8.3)	141 (9.5)	64 (12.1)	9 (6.0)	5 (3.9)	10 (6.5)
3	114 (14.4)	307 (13.8)	70 (11.1)	46 (13.2)	179 (12.0)	71 (13.5)	16 (10.6)	12 (9.4)	12 (7.8)
4	108 (13.7)	284 (12.8)	90 (14.3)	38 (10.9)	188 (12.7)	81 (15.4)	21 (13.9)	17 (13.4)	16 (10.4)
5	90 (11.4)	234 (10.5)	68 (10.8)	40 (11.5)	150 (10.1)	55 (10.4)	24 (15.9)	12 (9.4)	7 (4.5)
6	60 (7.6)	178 (8.0)	64 (10.2)	41 (11.8)	118 (7.9)	46 (8.7)	19 (12.6)	13 (10.2)	15 (9.7)
7	71 (9.0)	209 (9.4)	70 (11.1)	22 (6.3)	152 (10.2)	50 (9.5)	12 (7.9)	16 (12.6)	19 (12.3)
8	89 (11.3)	225 (10.1)	72 (11.5)	39 (11.2)	153 (10.3)	44 (8.3)	21 (13.9)	12 (9.4)	18 (11.7)
9	55 (7.0)	159 (7.1)	48 (7.6)	32 (9.2)	126 (8.5)	39 (7.4)	11 (7.3)	14 (11.0)	14 (9.1)
10	29 (3.7)	82 (3.7)	27 (4.3)	16 (4.6)	59 (4.0)	18 (3.4)	4 (2.6)	5 (3.9)	8 (5.2)
11	12 (1.5)	41 (1.8)	5 (0.8）	11 (3.2)	31 (2.1)	13 (2.5)	2 (1.3)	3 (2.4)	8 (5.2)
12	5 (0.6)	24 (1.1)	5 (0.8)	1 (0.3)	8 (0.5)	4 (0.8)	0 (0.0)	2 (1.6)	1 (0.6)
13	4 (0.5)	12 (0.5)	2 (0.3)	3 (0.9)	12 (0.8)	4 (0.8)	2 (1.3)	0 (0.0)	4 (2.6)
>13	24 (3.0)	69 (3.1)	7 (1.1)	14 (4.0)	54 (3.6)	15 (2.8)	7 (4.6)	5 (3.9)	9 (5.8)
Total (row%)	789 (12.3)	2226 (34.6)	628 (9.8)	348 (5.4)	1486 (23.1)	527 (8.2)	151 (2.3)	127 (2.0)	154 (2.4)

Similar bimodal distributions by hour of work were observed across different age groups, construction sectors, and occupations (data not shown). Industry and occupation were significantly associated with the hour of work (Chi‐squared *P*‐values: ≤.001), whereas the age group was not statistically significant (Chi‐squared *P*‐value: .36).

### 3.4 Medical cost and lost work days

Injury severity was measured by compensated medical cost and lost work days in this study. The median compensated medical cost involved in a claim was approximately $5243 and the average compensated medical cost was approximately $12 000, with a minimum of $8 and a maximum of $417 239 (hereinafter, in 2013 US dollars). The compensated lost work days due to the injury or illness ranged from 0 to 1696 days, with a mean of 80.5 days.

Injuries that happened in 2009 and 2010 were the most severe as indicated by higher average compensated medical cost and more average lost work days (Table 1). Claims from 2013 had much lower medical cost and less lost work days over all the years. Both medical cost and lost work days increased consistently with the workers’ age. Compared with workers aged 25‐34, the average medical cost for young workers aged 16‐24 was approximately $3500 lower, and average lost work days 24 days less. Older age groups had much higher medical cost and more lost work days. Medical cost and lost work days varied depending on the time of injury. For work shifts starting during evening and night time, the injuries were more severe. Similarly, injuries that occurred during evening and night time had higher average medical cost and more average lost work days.

Medical cost and lost work days were associated with injury nature. Minor injuries such as surface wounds and bruises had the lowest average medical cost of $3870, and the lowest average lost work days of 27.2 days. Traumatic injuries to bones, nerves, the spinal cord, and the occurrence of multiple injuries had more than $11 700 higher average medical cost, and approximately 80 more lost work days compared with surface wounds and bruises. Other traumatic injuries had an average medical cost as high as $31 799, and average lost work days of 205 days.

Injuries and illnesses that occurred from the 5th to 7th hour of work had higher medical cost and more lost work days, and those in the 13th hour had substantially high medical cost and more lost work days (Figure 1). The pattern of injury severity tended to inversely match the pattern of injury frequency, especially within the first 8 hours. It is worth noting that injuries occurring in the first hour of work (the very beginning time of a work shift) were also relatively more severe compared with the initial few hours.

Multiple linear regression models identified a statistically significant association between hour of work and medical cost (*P*‐value: .03) but not between hour of work and lost work days (*P*‐value: .44), adjusted for age, gender, construction sector, occupation, injury nature, weekly wage, and injury time. The 5th and the 13th hour of work had significantly higher medical costs compared with the first hour of work (adjusted Relative Medians (RMs): 1.21 and 1.60; 95% CI: 1.02‐1.44 and 1.01‐2.53, respectively; Table 3).

**Table 3:** Associations of different hour of work with compensated medical cost, Oregon construction industry, 2007‐2013^a^

Hour of work	Relative median (95% CI)
1st h	1
2nd h	0.90 (0.76, 1.08)
3rd h	1.04 (0.88, 1.24)
4th h	1.01 (0.86, 1.20)
5th h	1.21 (1.02, 1.44)
6th h	1.12 (0.93, 1.34)
7th h	1.12 (0.94, 1.34)
8th h	1.04 (0.87, 1.23)
9th h	1.05 (0.87, 1.23)
10th h	1.05 (0.84, 1.32)
11th h	1.00 (0.75, 1.34)
12th h	0.76 (0.50, 1.17)
13th h	1.60 (1.01, 2.53)
>13 h	1.10 (0.85, 1.42)

Abbreviation: CI, confidence interval.

^a^ Linear regression models (N = 6134), adjusting for: age, gender, construction sector, occupation, weekly wage, injury nature, and injury time.

Sensitivity analyses showed no meaningful difference in results with and without the 2013 data.

## 4 DISCUSSION

This study analyzed WC accepted disabling claims for the construction industry in the State of Oregon in the US from a 7‐year period of 2007‐2013. Injury frequency distribution, injury rate, medical cost and lost work days by year, demographics, employment, injury nature, and temporal factors in the Oregon construction industry were reported. This study also contributes to the limited body of literature on the distribution and severity of injuries and illnesses by hour of work.

From 2007 to 2013, both the count and rate of accepted disabling WC claims in the Oregon construction industry declined. This is in line with the national declining trend of injuries in the construction industry over this time period.^14^ This declining trend in the Oregon construction industry is also consistent with the trend of all‐industry accepted disabling claims in Oregon during this study period. However, the claims rate in the construction industry remains more than doubled compared with the all‐industry rate (average annual rate: 2.21 per 100 workers vs 1.02 per 100 workers).^10^

Most disabling injuries and illnesses happened to male construction workers. This is conceivable as the construction industry is dominated by male workers.^14^ Furthermore, this study showed that the injury rate in male workers was more than five times the rate in female workers. Injury rate was also significantly higher among young workers. Male workers and young workers should be the target of injury prevention intervention in the construction industry, in terms of reducing injury risk. Older workers had higher medical cost and lost work days, indicating they suffered more negative health impact from the work‐related injury or illness. Our findings that injuries and illnesses occurring at night or with a work shift starting at evening and night time had higher medical cost and more lost work days suggest shift workers tended to have more severe work‐related accidents.

It is noteworthy that the building foundation and exterior contractor subsector had an injury rate over 50% higher than the average rate, and more than double the lowest injury rate among subsectors. Other subsectors such as heavy and civil engineering construction also had a relatively higher injury rate. The construction industry has long been regarded as one of the most dangerous industries with high fatal and non‐fatal accident rates. Construction workers are often exposed to dangerous working conditions such as working at heights or climbing ladders and scaffolds. They often operate hazardous equipment and may be exposed to various physical and chemical agents such as noise and dust.^15^, ^16^, ^17^ Depending on the varied job tasks, workers in some subsectors may be exposed to a more hazardous environment, resulting in higher injury and illness risk. For instance, reports have shown that the building foundation and exterior contractor subsector had a very high percentage of workers (49.4%) exposed to silica compared with other subsectors, and the percentage of workers exposed at a silica level of 250 μg/m^3^ was the highest (10%).^1^ In addition, our data indicate that this subsector had the highest percentage of male workers (98%) and young workers (16%). More research investigating the factors leading to high injury rates in the construction industry in general as well as in certain subsectors, such as the building foundation and exterior contractors, is needed to inform effective prevention intervention strategies for worker safety and health.

The study adds information on injury distribution by hour of work. We found a bimodal distribution of injury and illness by working time, which is consistent with findings reported in previous research.^6^, ^7^, ^18^ Our findings are consistent with a study on Oregon WC data for the construction industry from 1990 to 1997, which found that the highest number of injuries occurred in the 3rd hour of work.^8^ Previous studies attributed the similar bimodal distribution to the effect of a meal break.^18^ This is plausible for Oregon construction workers. Oregon state law requires a meal break of at least 30 minutes with relief from all duties between the 3rd and 6th hour of work for a work period of 8 hours, and a minimum 10‐minute rest period every 4‐hour segment.^19^, ^20^ Because most construction workers began work between 6:00 and 8:00 in this study, the distribution matches the time frame of a meal break. Interestingly, our study shows that digestive diseases happened more frequently in the 5th and 6th hour of work. The reduced number of injuries during the 5th to 7th hour of work might be related to the restorative effect of meal and rest breaks. Rest breaks have the potential to help workers recover from fatigue accumulated over work, which may impact injury risk.^21^, ^22^, ^23^ Tucker, Folkard, and Macdonald^24^ found in the manufacturing industry that injury risk decreased after a break. The authors concluded that rest breaks successfully counteracted the accumulation of fatigue and injury risk. More recent studies have also shown that rest breaks significantly delayed the onset of injuries.^25^, ^26^

Medical cost and compensated lost work days are often used as indices for injury severity.^18^, ^27^, ^28^ An important finding in this study is that hours in the middle of an 8‐hour work shift (particularly the 5th hour of work) and the 13th hour of work were significantly associated with more severe injuries and illnesses after adjusting for factors that were statistically associated with medical cost. No previous study has reported on the relationships between certain hours of work and injury severity to the best of our knowledge. Further research is needed to investigate the pattern of rest breaks by construction workers during work, their workload at the time of the injury, and work‐related fatigue to better understand possible causes and contributing factors for more severe injuries in these hours. Although the lost work days is also an often used proxy for injury severity in existing literature, no statistically significant association was found between hour of work and lost work days in our study. We noted that the compensated lost work days recorded in the Oregon workers’ compensation data may not reflect the actual days of lost work due to a 3‐day waiting period and programs facilitating workers return to work sooner.^9^, ^29^ Further research could investigate whether similar association exists between hour of work and true lost work days.

A few limitations should be acknowledged. Only accepted disabling WC claims were included in this study, which represent the most severe work‐related injuries and illnesses. WC claims data are often subject to under‐reporting and under‐representativeness of work‐related injuries and illnesses.^10^, ^30^, ^31^, ^32^ However, the WC claims data have detailed information on injury nature and temporal factors such as work shift time, which made this study possible.

Caution must be taken in the study's interpretation due to the limitations of the WC disabling claims dataset used in this study. As noted before, the number of WC claims in 2013 was underestimated by 21% in this study, driving an overall underestimation rate of 3% for the study period. Furthermore, recording cost‐related information associated with WC claims is always a dynamic process. As medical cost and lost work days accrue in future years when treatment continues or disease status changes, the claims will be re‐opened to update the information. As such, underestimation of medical cost and lost work days is a constant issue in any given dataset, but this issue is particularly evident for claims with less mature time. By comparing with more recent WC data in DCBS, the authors learned that the significant decrease in average medical cost and lost work days in 2013 reflects largely underestimation, instead of an actual decreasing trend. The underestimation in 2013 may slightly impact medical cost and lost work days estimates for the whole study period. However, we did not find meaningful difference in the results after removing the 2013 data.

A strength of this study is that the hour of work as operationalized in this WC claims dataset precludes recall bias compared with other studies that have used self‐reported working hours.^33^, ^34^, ^35^ However, the recording of hour of work in the WC claims information may have limitations. Because hours spent at work was not directly recorded in the dataset, we used the difference between the shift starting time and the injury time as a proximate alternative. This may be subject to misclassification, particularly when the calculated hour of work exceeds 12 hours, the generally suggested maximum daily working hours.^36^

The identified trends and patterns of work‐related injuries and illnesses in Oregon construction industry can be generalized only with caution. Further research is needed to investigate whether similar associations exist between hour of work and injury frequency and severity in other regions and industries.

## 5 CONCLUSION

This study reported on injury frequency and rate by year, demographics, employment, and temporal factors in the construction industry by analyzing the Oregon WC accepted disabling claims data from 2007 to 2013. Compensated medical cost and lost work days were examined to indicate injury severity. Oregon construction workers suffered a higher than all‐industry injury rate, although both the injury count and rate declined through the study period. Male workers and young workers had the highest injury rates compared with other gender and age groups, whereas older workers had more severe injuries and illnesses. Injuries and illnesses that occurred at night were more severe.

The study is among few studies examining injury frequency by hour of work and the first to investigate the association between hour of work and injury severity to the best of our knowledge. The study showed a bimodal distribution of work‐related injuries over successive working hours among construction workers. Hours in the middle of a regular 8‐hour work shift and the 13th hour of work were associated with significantly higher medical cost.

The study has practical implications to injury prevention interventions. While further research is needed to understand the specific factors causing more severe injuries and illnesses in the mid‐shift work periods, findings in this study reflect the importance of raising awareness for interventions targeting certain subpopulations defined by demographics and employment, and specific working time periods including the mid‐shift and end‐shift work periods to improve safety and health for construction workers.

## DISCLOSURE

*Approval of the research protocol*: N/A. *Informed consent*: N/A. *Registry of the study/trial*: N/A. *Animal studies*: N/A. *Conflict of interest*: The authors declare no conflict of interests for this article*.*

## AUTHOR CONTRIBUTIONS

LY substantially worked on conception and design of the study, analysis and interpretation of data, and writing of the manuscript. LK guided the study. AB, LK, and ES substantially contributed to analysis and interpretation of data. KH and DD substantially contributed to the acquisition and interpretation of data. All authors worked on revising of the manuscript critically for important intellectual content and final approval to be published.
